# Vaccines against human papillomavirus in low and middle income countries: a review of safety, immunogenicity and efficacy

**DOI:** 10.1186/s13027-015-0012-2

**Published:** 2015-06-12

**Authors:** Miriam Nakalembe, Florence M. Mirembe, Cecily Banura

**Affiliations:** Department of Obstetrics and Gynaecology, Makerere University, Kampala, Uganda; Department of Child Health and Development Center, Makerere University, Kampala, Uganda

**Keywords:** Human papillomavirus vaccines, Immunogenicity, Safety, Efficacy, Low middle income countries

## Abstract

Currently, there is limited data on the immunogenicity and efficacy of human papillomavirus vaccines in Low and Middle income countries (LMIC). The review aims to summarize the current status from published HPV vaccine safety, immunogenicity and efficacy studies in low and middle income countries (LMIC). Electronic databases (PubMed/MEDLINE and HINARI) were searched for peer reviewed English language articles on HPV vaccination in LMIC that have so far been published from 1st January 2006 up to 30th January 2015. Eligible studies were included if they had used the bivalent (bHPV) or quadrivalent HPV (qHPV) vaccines in a LMIC and investigated safety, immunogenicity and/or efficacy. The main findings were extracted and summarized. A total of fourteen HPV vaccine studies assessing safety, Immunogenicity and efficacy of the bivalent or quadrivalent vaccines in LMIC were included. There are only ten published clinical trials where a LMIC has participated. There was no published study so far that assessed efficacy of the HPV vaccines in Sub-Saharan Africa. From these studies, vaccine induced immune response was comparable to that from results of HICs for all age groups. Studies assessing HPV vaccine efficacy of the bivalent or quadrivalent vaccine within LMIC were largely missing. Only three studies were found where a LMIC was part of a multi center clinical trial. In all the studies, there were no vaccine related serious adverse events. The findings from the only study that investigated less than three doses of the bivalent HPV-16/18 vaccine suggest that even with less than three doses, antibody levels were still comparable with older women where efficacy has been proven. The few studies from LMIC in this review had comparable safety, Immunogenicity and efficacy profiles like in HIC. Overall, the LMIC of Africa where immune compromising/modulating situations are prevalent, there is need for long term immunogenicity as well as surveillance studies for long term clinical effectiveness after two and three dose regimens.

## Background

Globally, cervical cancer is the 3rd most common malignancy among women with more than 530,000 incident cases and over 275,000 deaths annually [1]. The greatest burden of disease (over 85 %) occurs in the LMIC where there is lack of or limited organized screening and treatment programs that are present in the high income countries [2].

Human Papilloma Virus (HPV), the most common sexually transmitted infection has been recognized as a necessary cause of cervical cancer [3]. There are over 100 types of HPV genotypes, with HPV types 16 and 18 being responsible for approximately 70 % of cervical cancers worldwide, and types 6 and 11 for 96–100 % of genital warts infections [4]. Besides cervical cancer, certain HPV types are also associated with a proportion of cancers of the anus, oropharynx, the vulva, vagina and the penis which may too be impacted by the HPV vaccines [5]. However, the incidence rates of these cancers are much lower (e.g., estimated global incidence for anal cancer is 1 per 100,000 with 27,000cases per year), [5] than that of cervical cancer and the HPV vaccines impact on their incidence remains to be known.

Prophylactic vaccines are composed of virus like particles made with synthetic L1 proteins derived from HPV 6, 11, 16 and 18 that assemble together to form an empty virus-like capsid. The bivalent (Cervarix™) that protects against HPV 16 and 18 and the quadrivalent (Gardasil™) that protects against HPV 6, 11, 16 & 18 have been approved in 2007 and 2009, respectively [6, 7]. Both vaccines protect against infections and lesions induced by the HPV vaccine types, provided that the recipient has not been exposed to these HPV types before vaccination. Availability of the prophylactic human papillomavirus (HPV) vaccine is seen as a key strategy in reducing the burden of cervical cancer in low and middle income countries (LMIC) where this has so far been unachievable [8].

To date, at least 110 countries have licensed the bivalent HPV vaccine and over 120 countries have licensed the quadrivalent vaccine [9]. However, despite the licensure, about 51 countries worldwide have incorporated the vaccine in their National vaccination programs and only six countries are LMIC (85 countries comprise LMIC as per World Bank list (July 2014) [10]. WHO recognizes the global importance of preventing cervical cancer and HPV-related diseases and therefore recommends that HPV immunization should be part of the national immunization programs where countries meet certain criteria [11]. There is no doubt that in LMIC, cervical cancer an HPV related-disease, constitutes a public health problem. However, many LMIC have limited resources for introducing a new vaccines especially one like the HPV whose impact may not be seen immediately when compared to those against major childhood diseases like the rotavirus and pneumococcal vaccines [12]. In addition, significant financial and political barriers still exist as well as programmatic challenges of delivering a vaccine to adolescents in settings where school attendance is low or irregular [9]. Despite the challenges, about twenty six of the LMIC have completed or are currently engaged in HPV vaccine piloting activities in order to inform decision making for future national rollout of these vaccines [9]. According to studies that have assessed HPV vaccine implementation in LMIC, high rates of acceptability and coverage have been achieved through various delivery strategies and the lesson learned have been documented and shared [9, 13]. The challenge posed by financial barriers has been overcome in some of the LMIC thorough partnerships with the manufacturers of the vaccines and the Global Alliance for Vaccine Initiative (GAVI), therefore, many low-income countries have the opportunity to incorporate the HPV vaccine in their national vaccination programs.

Even as countries continue to roll out HPV vaccination as a primary strategy to prevent cervical cancer, it will probably be over 20 years before the impact of the vaccine on cervical cancer is realized. Yet in the LMIC, data on safety, immunogenicity, efficacy and long term follow up is still sparse [14].

In this review, we summarize the current status of HPV vaccine safety, immunogenicity and efficacy studies available in LMIC countries and discuss the findings in the context of ongoing advances in HPV vaccine development. We discussed the findings and identified gaps for future research in these countries.

## Review

### Methods

To review the current status of HPV vaccine safety, Immunogenicity and efficacy studies in LMIC, electronic databases (PubMed/ MEDLINE and HINARI) were searched for peer reviewed English language articles on HPV vaccination in LMIC that have so far been published from 1st January 2006 up to 31st January 2015. Search terms included HPV vaccination and the name of a LMIC as per World Bank list (July 2014) [10]. Studies were eligible if they were HPV vaccine studies in a LMIC or a LMIC was included in a multi center study. In addition, clincalTrials.gov was searched for unpublished HPV vaccine studies that were ongoing, stopped or withdrawn.

The titles and abstracts of the articles were reviewed for inclusion. Articles were included if they had used the bivalent or quadrivalent HPV vaccine in a LMIC and investigated safety, immunogenicity and or HPV disease end points. Studies on HPV vaccine delivery, knowledge, attitudes and practice around HPV vaccines, opinions about HPV vaccines were excluded. Information was abstracted from each article on: the first author & year of publication, country of study, study design, study population, vaccine type, schedule of administration, duration of follow up, serological tests to measure the immune response and PCR assays to detect HPV DNA. We also abstracted information of safety-(unsolicited and solicited symptoms), immunogenicity (antibody titers) and efficacy (based on virological and disease end points) of the bivalent or quadrivalent vaccines among the populations who were studied.

## Results

In total, 467 studies were found on searching the databases on published studies of HPV vaccines in LMICs. On searching the titles and abstracts, fourteen HPV vaccine studies were indentified that reported on immunogenicity, efficacy (both summarized in Table 1) and safety (summarized in Table 2) of the HPV vaccines. Ten (10) were randomized controlled clinical trials; five double blinded, three open label with one cluster randomized, two partially blinded. There were two sub studies (longitudinal observational studies) nested within a phase IIIb clinical trial, three cross sectional studies (follow up of students in a demonstration project). The studies were conducted in Latin America (Honduras, Colombia, Guetamala), Asia (India, Vietnam, Phillipines, Bangladesh), and Africa (Senegal, Tanzania and Uganda). From ClinicalTrials.gov, a total of 159 registered ongoing or completed HPV vaccine clinical trials were identified out of which 18 were from a LMIC; ten published, six ongoing or completed, two withdrawn and another suspended.

**Table 1:** Summary of vaccine immunogenicity and efficacy studies conducted in low and middle income countries

Study	Study population	Study vaccine	Study design	Vaccination schedule	Follow-up	Serological assays/HPV DNA assay	Study end points	Main results
Perez et al. (2008) [15]	A total of 6004 healthy female subjects aged 9–24 were recruited from Brazil, Mexico, Colombia, Costa Rica, Guatemala and Peru	Quadrivalent HPV vaccine	Randomised blinded controlled trial	0,2,6 months	7 months	cLIA	Geometric mean anti-HPV-6, 11, 16 and—18 antibody titres. Positivity to HPV 6, 11, 16 and 18 by PCR and CIN lesions. Any Condyloma VIN 1 or VaIN	The vaccine was 92.8 and 100 % effective in preventing cervical intraepithelial neoplasia and external genital lesions related to vaccine HPV types, respectively.
Muñoz et al. (2009) [25]	Multi-center trial Colombia, France, Germany, Philippines, Spain, Thailand, and the USA. Healthy Women aged 24–45 years *n* = 3819	Quadrivalent HPV vaccine	Randomised, placebo-controlled, double-blind study	At 0, 2 and 6 months	Mean follow up 2.2 years	HPV multiplex PCR testing	Incidence of cervical and external genital disease related to HPV 6, 11, 16, or 18; and to HPV 16 or 18 alone	The quadrivalent vaccine is effi cacious in women aged 24–45 years not infected with the relevant HPV types at enrolment.
Medina et al. (2010) [16]	Multi center with Healthy Honduras girls 10–26 years *n* = 2067	Bivalent HPV-16/18 vaccine	Randomised controlled observer blind trial (1:1)	at 0, 1, and 6 months	7 months	ELISA	Safety and immunogenicity	The vaccine was generally well tolerated and immunogenic among these girls.(GMT HPV-16 14,778.0 (95 % CI 12,668.5-17,238.7 and HPV 18 6149.1 (95 % CI 5314.5- 7114.7)
Bhatla et al. (2010) [19]	Healthy Indian women of an older age group 18–35 years (*n* = 330)	Bivalent HPV-16/18 Vaccine	Double blinded randomized multi center trial	at 0, 1, and 6 months	7 months	VLP ELISA	Geometric mean anti-HPV-16 and −18 antibody titres	Vaccine was highly immunogenic and safe in this older population of women (GMT levels HPV-16 10226.5 (95 % CI 8847.1-11821.0) and HPV-18 3953.0 (95 % CI 3421.8-4566.8)
Neuzil et al. (2011) [27]	Adolescent Vietnam girls 11–13 year *n* = 903	Quadrivalent HPV	Open-label, cluster randomized, noninferiority study	3 doses of quadrivalent HPV vaccine on a standard dosing schedule (at 0, 2, and 6 months) and 3 alternative dosing schedules (at 0, 3, and 9 months; at 0, 6, and 12 months; or at 0, 12, and 24 months.	1 month after receipt of the third vaccine dose	cLIA	Geometric mean anti-HPV-6, 11,16 and −18 antibody titres	Vaccine administration on standard and alternative schedules was immunogenic and well tolerated. Use of alternative dosing compared with a standard schedule did not result in inferior antibody concentrations.
Salif Sow et al. (2012) [14]	African girls (Tanzanian and Senegalese) girls and young women, seronegative for human immunodeficiency virus (HIV) 10–25 years (*n* = 450), placebo (*n* = 226)	HPV-16/18 AS04-adjuvanted vaccine	Double blinded, randomized controlled (2:1)	at 0, 1, and 6 months	Up to 12 months	VLP ELISA	Geometric mean anti-HPV-16 and −18 antibody titers	The vaccine was highly immunogenic with 100 % seropositive for both anti–HPV-16 and anti–HPV-18 antibodies and safety profile
Khatun et al. (2012) [18]	Healthy adolescent Bangladesh girls 9–13 years *n* = 67	Bivalent HPV-16/18 vaccine	Randomized controlled trial (3:1)	0, 1, 6 months	7 months	ELISA	Geometric mean anti-HPV-16 and −18 antibody titres	Vaccine was well tolerated, and highly immunogenic
Schwarz et al. (2012) [17]	Healthy Girls 10–14 years from 31 centers in Taiwan, Germany, Honduras, Panama, and Colombia	HPV-16/18 AS04-adjuvanted	Open label randomized trial	at 0, 1, and 6 months	Four year follow up	ELISA	Geometric mean anti-HPV-16 and −18 antibody titres	The HPV-16/18 AS04-adjuvanted vaccine produces anti-HPV-16 and anti-HPV-18 antibody titers that are maintained for up to 4 years at higher levels than those in young women in whom vaccine efficacy against cervical lesions was demonstrated.
	*n* = 1035							
Brown et al. (2013) [22]	Tanzanian females 10–25 years (*n* = 298)	HPV-16/18 AS04-adjuvanted vaccine	Sub-study nested within a Phase IIIb immunogenicity and safety trial of the HPV-16/18 AS04-adjuvanted vaccine	at 0, 1, and 6 months	Follow up to 12 months	ELISA	Geometric mean anti-HPV-16 and −18 antibody titers	Parasitic infections were common overall, the vaccine induced high HPV-16/18 GMTs, (HPV 16 10,786 EU/mL (95 % CI 9126–12,747), and HPV-18 3701 EU/mL (95 % CI 3156–4340). There was no evidence of a reduction in HPV-16 or HPV-18 GMT at Month 7 or Month 12 follow-up visits among participants with helminths or malaria.
LaMontagne et al. (2013) [28]	Vietnam girls 11–13 year *n* = 903	Quadrivalent HPV vaccine	Open-label, cluster randomized, noninferiority study	3 doses of quadrivalent HPV vaccine on a standard dosing schedule (at 0, 2, and 6 months) and 3 alternative dosing schedules (at 0, 3, and 9 months; at 0, 6, and 12 months; or at 0, 12, and 24 months.	>2 years follow up	cLIA	Geometric mean anti-HPV-6, 11,16 and −18 antibody titres.	HPV vaccine dose- timing, and extended schedules do not produce inferior immune responses. In addition, 2 doses of HPV vaccine delivered at 0 and 12 months might afford similar protection
Nakalembe et al. (2014) [21]	Ugandan girls 10–16 years	HPV-16/18 AS04-adjuvanted vaccine	Cross-sectional study follow up on girls vaccinated in 2010 in an HPV demonstration project	0,1 and 6 months	18 months post vaccination	Multiplex HPV serology	Median Flourescent intenstity	The AS04-Adjuvanted HPV-16/18 vaccinated girls showed a higher level of antibodies to HPV-16/18(HPV-16 4691 95 % CI: 4438–4958 among the vaccinated compared to 218 95 % CI: 190–252 among the unvaccinated girls; HPV-18 1326 95 % CI: 1470–1776 among the vaccinated compared to 103 95 % CI: 88–121) among unvaccinated girls) and other non-vaccine hrHPV types compared to the unvaccinated girls.
	(*n* = 404)							
LaMontagne et al. (2014) [29]	Ugandan girls 10–17 years	HPV-16/18 AS04-adjuvanted vaccine	Cross-sectional follow-up study. Girls vaccinated in a government-run HPV vaccination demonstration program	0,1 and 6 months	At month 36 post vaccination	VLP ELISA	Geometric mean anti-HPV-16 and −18 antibody titres	The immunogenicity with less than three doses did not meet a priori non-inferiority thresholds. However, antibody levels measured ≥24 months after last dose were similar to those of adult women where efficacy has been demonstrated (GMTs HPV161-dose = 230 HPV16 2-dose = 808,and HPV16 3-dose = 1607; HPV181-dose = 87, HPV182-dose = 270, and HPV183-dose = 296 EU/mL) The GMT ratio for 2:3 doses was 0.50 for HPV16 and 0.68 for HPV18).
	One dose *n* = 230, 2 doses *n* = 808, 3 doses *n* = 1608							
Skinner et al. (2014) [26]	Phillipines >25 year old women *n* = 5752	HPV-16/18 AS 04-adjuvanted vaccine	double-blind, randomised controlled trial	At 0, 1 and 6 months	Mean follow up 40.3 months	HPV DNA	6-month persistent infection with HPV 16 or HPV 18 (HPV 16/18) or CIN grade 1 or greater (CIN1+) associated with HPV 16/18.	The HPV 16/18 vaccine is efficacious against infections and cervical abnormalities associated with the vaccine types, as well as infections with the non-vaccine HPV types 31 and 45.

**Table 2:** Summary of the safety endpoint data of HPV vaccines in low and middle income countries

Study	Sample	Safety end point assessments	Results
Perez et al. (2008) (qHPV) [15]	Multicenter cohort vaccine *n* = 3147, placebo *n* = 2857	Reported adverse experiences that included injection site pain, swelling and erythema and any other systemic adverse experience as filled out on the adverse event card.	There was no significant difference in occurrence of serious adverse events between the two groups. However, more adverse experiences were reported by subjects who received quadrivalent HPV vaccine compared to those subjects who received placebo with occurrence of injection-site adverse experiences responsible for the increase in adverse experiences seen in these participants.
Muñoz et al. (2009) (qHPV) [25]	Multi-center cohort vaccine *n* = 1910, placebo *n* = 1907	Information about adverse events was gathered from participants by general questioning at study visits and by use of a vaccine report card. The participants received the card at every vaccination visit to record temperatures and local and systemic adverse events.	The proportion of participants who reported serious adverse event on day 1–15 after any vaccination was comparable between the two groups. Injection-site adverse events were mainly responsible for the slight increase in adverse events that were recorded in the vaccine group
Medina et al. (2010) (bHPV) [16]	Multi center cohort vaccine *n* = 1035, placebo *n* = 1032	Solicited local and general symptoms (pain, redness, swelling, fever, headache, fatigue, gastrointestinal symptoms, arthralgia, myalgia, rash, and urticaria) were reported for 7 days while unsolicited symptoms were reported for 30 days. The intensity of solicited and unsolicited symptoms was graded on a scale of 0–3. SAEs, NOCDs, medically significant conditions (MSCs), pregnancies and their outcomes were reported up to month 12. All solicited local AEs were considered related to vaccination. Other AEs (solicited general and unsolicited) were assessed for causality by investigators.	The occurrence of SAEs was similar in both groups. Between months 7 and 12, 13 girls (1.3 %) and 10 girls (1.0 %) reported SAEs in the HPV-16/18 vaccine and control groups, respectively. The pattern of symptoms was similar in both groups with respect to incidence, severity and duration. The incidence of local and solicited symptoms did not increase with the second and third vaccine doses.
Bhatla et al. (2010) (bHPV) [19]	Multicenter study vaccine *n* = 176, placebo *n* = 178	For 7 days after each dose, local symptoms (pain, redness and swelling at the injection site) and general symptoms (fever, headache, fatigue, gastrointestinal symptoms, arthralgia, myalgia, rash and urticaria) were solicited and recorded on diary cards. Each symptom was graded from 1 to 3 based on the extent of discomfort that was reported. Investigators actively solicited for any pregnancy information from the participants and confirmed by urine pregnancy tests prior to each vaccine dose. Unsolicited events were followed for 30 days after each vaccination. Serious adverse events (SAE), any new-onset chronic disorders (NOCD and other medically significant conditions (MSC) were followed up.	Solicited local injection site symptoms (pain, redness and swelling) were more frequent in the Vaccine group than the Placebo group. Solicited general symptoms (fatigue, headache and fever) were similar in both groups. There was no difference observed between the two groups for any unsolicited symptoms. Six SAE were reported during the study period, two in the Vaccine group (acute appendicitis and lymph node tuberculosis) and four in the Placebo group(bronchogenic cyst, cataract, a miscarriage and pneumothorax of the left lung) with none considered related to vaccination by the investigators. None of these SAE was fatal.
Neuzil et al. (2011) (qHPV) [27]	Longitudinal cohort alternate dose schedules *n* = 903. Events recorded up to 30 days after last dose	Solicited adverse events (local reactions and fever) and unsolicited adverse events were recorded. All serious adverse events occurring up to 30 days following the last dose of vaccine were documented.	The vaccine was generally well tolerated in each dosing schedule group. Solicited and unsolicited adverse events following any vaccination were comparable across groups. Pain at the injection site was the most common adverse event in all groups with most episodes classified as mild. No serious adverse events occurred within the 30 days of each vaccination. Throughout the study, there were no deaths, vaccine-related serious adverse events reported.
Salif Sow et al. (2012) (bHPV) [14]	Randomised cohort (vaccine *n* = 450/ 1298 doses, placebo *n* = 226), 0–12 months period	Solicited and unsolicited local symptoms (pain or swelling at injection site) and general symptoms (arthralgia, fatigue, fever, gastrointestinalsymptoms, headache, myalgia, rash, or urticaria). Grade 3 symptoms defined as swelling at the injection site >50 mm in diameter, fever >39 °C (axillary), urticaria distributed on ≥4 body areas, as well as other symptoms that prevented normal daily activity. Serious AEs (SAEs), other medically significant conditions.	There were no vaccine related serious adverse events and no participant withdrew due to an adverse event. However, the incidence of any solicited symptom was higher for vaccine recipients than for placebo due to a higher incidence of local symptoms.
Khatun et al. (2012) (bHPV) [18]	Randomised cohort vaccine *n* = 50, placebo *n* = 17	Local symptoms (pain redness and swelling at the injection site) as well as general symptoms(fever, headache, fatigue, gastrointestinal symptoms that included nausea, vomiting, diarrhea and/or abdominal pain, arthralgia, myalgia, rash and urticaria) were assessed for five consecutive days after each dose. The intensity of each symptom was graded on a non-quantifiable scale from mild, moderate and severe based on the extent of discomfort. Unwanted events were followed for 14 days after each vaccination.	The vaccine was well tolerated with no reports of serious vaccine-related adverse experiences between enrollment and Month. Fever and injection site pain were the most frequent though most were mild among the vaccinated group.
Watsone-Jones et al. (2012) (qHPV) [44]	Cluster-randomized trial 134 primary schools randomly assigned to class-based (school grade [class] 6) or age-based (girls born in 1998; 67 schools per arm) vaccine delivery *n* = 5532	Vaccinees requested their parents to call in the event of any suspected adverse event (AE) and to go to the nearest health facility. Adverse events were also recorded at each school visit. SAEs or AEs that indicated potential vaccine reactions were investigated by a senior clinician	Vaccine-related adverse events were rare. There were 11 AEs reported with 3 SAEs not thought to be related to the vaccine. The vaccine was generally acceptable and safe [43]
Schwarz et al. (2012) (bHPV) [17]	Four year follow up. Total vaccinated cohort (TVC) *n* = 617	Serious adverse events, new onset chronic diseases (NOCDs), medically significant conditions, and pregnancies were recorded in the follow-up month 48. An event was considered a potential NOCD if there was no record of it in the participant history. Analysis of AEs incidence rate per 100,000 subjects per year was performed.	No participants withdrew from the study because of an AE, and there were no fatal events. There were no vaccine related SAEs. There was no apparent difference in terms of incidence rates of AEs between the study groups in the cumulative follow-up time. A total of 32 pregnancies were reported throughout the study period in HPV-16/18 vaccine recipients with 29 participants giving birth to healthy babies.
Skinner et al. (2014) (bHPV) [26]	Multicenter cohort vaccine *n* = 2881, placebo *n* = 2241. Four year follow-up.	Solicited symptoms for 7 days and unsolicited symptoms for 30 days after each vaccination were recorded by participants. Serious adverse events, new-onset chronic diseases, new-onset autoimmune diseases, medically significant conditions, pregnancy, and pregnancy outcomes were recorded throughout the 48-month follow-up.	Solicited injection-site symptoms and other general solicited symptoms occurred more in the vaccine group than in the control group. Overall, the incidence of unsolicited symptoms, serious adverse events, medically significant conditions, new-onset chronic disease, and new-onset autoimmune disease was similar in both groups, and pregnancy outcomes did not differ between groups. There were seventeen deaths that occurred, 14 (<1 %) of 2881 women in the vaccine group and three (<1 %) of 2871 in the control group. None of these deaths was believed to be related to vaccination with no cluster of disease type noted.

### Immunogenicity of the HPV vaccines

Perez et al. [15] in Guetemala conducted a multi center placebo controlled clinical trial that recruited 6004 female participants within age range of 9–24 years. Participating countries included Brazil, Mexico, Colombia, Costa Rica, Guatemala and Peru. Participants were randomized into two arms; immunization with intramuscular (deltoid) injections of the quadrivalent HPV vaccine or placebo. Procedures were carried out at enrollment (day 1), month, 2 and month 6. In this study, the participants were stratified according to age; 9–15, 16–24. At month 7, the 9–15 years old girls had a stronger antibody responses (HPV 6, 982.9 (95 % CI: 872.4−1107.5); HPV 11, 1242.7 (95 % CI: 1094.4−1411.3); HPV 16, 5163.9 (95 % CI: 4449.8−5992.7); HPV 18, 1036.5 (95 % CI: 890.1–1207.0) compared to those 16–24 years (HPV 6, 525.0 (95 %CI 502.6–548.4), HPV 11 730.9 (95 % CI 695.7–767.8), HPV 16 2540.3 (95 % CI: 2379.5–2711.9); HPV 18, 473.7 (95 % CI: 448.0–500.8) [15]. The results of the study showed that the vaccine was highly immunogenic in these participants.

Medina et al. [16] published the initial immunogenicity results of a phase III partially blinded, randomized, placebo controlled trial among adolescent girls (mean age 12 years) from 12 countries including Honduras (57 centers located in Australia, Colombia, the Czech Republic, France, Germany, Honduras, Korea, Norway, Panama, Spain, Sweden, and Taiwan). In this study, the girls received the AS04-adjuvanted HPV-16/18 vaccine or hepatitis A virus (the control arm) at 0, 1, and 6 months. The vaccine was immunogenic (GMT HPV16, 14,778.0 (95 % CI: 12,668.5–17,238.70) and HPV- 18, 6149.1 (95 % CI 5314.5–7114.7) enzyme-linked immunosorbent assay units (EU/mL) in initially seronegative girls from Honduras [16]. Later, Schwarz et al. [17] published follow-up data on the long-term immunogenicity of the vaccine among 10–14 years adolescent girls in the same cohort at month 48. All participants remained seropositive for anti-HPV-16 and anti-HPV-18 at month 48. It was observed that antibodies peaked at month 7 and then gradually declined into a plateau from month 18 to 24 onwards (GMTs at month 48; HPV–16, 2374.9 (95 % CI: 2205.7–2557.0) EL.U/mL and HPV- 18, 864.8 (95 % CI: 796.9–938.4) EL.U/mL) [17].

Schwarz et al. [18] followed up the same cohort of girls who participated in the study above (Schwarz et al. [17]) and found that up to 72 months, the vaccine induced anti-HPV-16 and −18 antibody levels remained high (GMTs HPV-16, 1962.0 EU/mL (95 % CI 1811.3–2125.3) and HPV-18, 749.6 EU/mL (95 % CI: 687.7–817.0) [18].

Anti-HPV-16 and −18 antibody GMTs at month 72 in this study were 65.8- and 33.0-fold higher than those induced by natural infection, respectively [18]. Further, based on a statistical model, it was predicted that the antibodies may remain above those induced by natural infection for over 20 years [17].

Similar immunogenicity results were realized from a randomized controlled trial using the bivalent HPV-16/18 vaccine among healthy Bangladesh girls 9–13 years (*n* = 67) by Khatun et al. [18]. The participants were followed up to 7 months from 1st vaccine dose. In this study, a commercial ELISA kit detecting HPV IgG antibodies to HPV-16/18 was used to detect seropositivity to HPV-16/18 with a cut-off value of 450 nm calculated from the mean optical density (OD) of the negative control [18]. Notwithstanding a small sample size, and a different HPV antibody assay, the sero-conversion rate was 97.5 % [18] as opposed to 100 % in the previous study of Schwarz et al. [17].

**Fig. 1:**
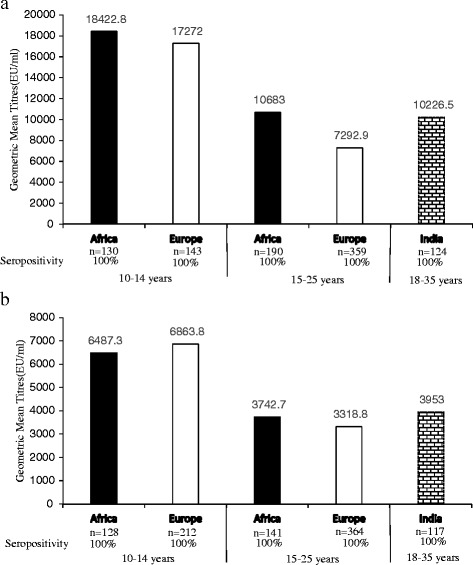
**a** & **b** Showing comparable Geometric mean titers for anti–HPV-16/18 at month 7 in 3 immunogenicity studies Africa [14], India [20] and Europe [24] across the age strata. In the vaccine groups for all the three studies, seropositivity to anti-HPV-16/18 was 100 % in initially seronegative participants

Bhatla et al. [19] assessed the immunogenicity of the bivalent vaccine among healthy Indian women of an older age group 18–35 years (*n* = 330) up to month 7. They also found the vaccine to be highly immunogenic in this older population of women (Fig. 1) [19].

In the LMIC of Africa, studies that addressed immunogenicity with the three dose bivalent HPV-16/18 vaccine were conducted in Tanzania, Senegal and Uganda. Sow et al. [14] published results from a multi-center placebo-controlled trial in Tanzania and Senegal among girls (age 10–14; 15–25 years) (*n* = 676). Girls were randomized to receive HPV-16/18 AS04-adjuvanted vaccine (*n* = 450) or placebo (*n* = 226) at 0, 1, and 6 months and followed up to 12 months post 1st vaccine dose. Similar to the studies above, girls < 15 years had a higher immune response than girls aged ≥15 years at month 7 month [14]. In this study, the HPV-16/18 AS04-adjuvanted vaccine was highly immunogenic and safe among HIV–seronegative African girls and young women (Fig. 1) [14].

In the same cohort, Brown et al. [20] conducted a sub-cohort study among the Tanzanian girls (*n* = 298) to measure the impact of malaria infections and helminth infestation on the immunogenicity of the bivalent HPV-16/18 vaccine. They found that parasitic infestations were common and that there was no evidence of a reduction in HPV-16 or HPV-18 GMT at Month 7 or at month 12 follow-up visits among participants with helminths or malaria [20]. However, it was observed that participants with malaria had increased GMTs though the mechanism and significance for the increase in GMT in those with malaria was not clearly understood. In Uganda, Nakalembe et al. [21] conducted a comparative cross sectional study at month 18 month of vaccination among 10–16 year old Ugandan students (*n* = 211) who had been vaccinated with the bivalent HPV vaccine in a demonstration project. At this time point, the AS04-Adjuvanted HPV-16/18 vaccinated girls showed a higher level of antibodies to HPV-16/18 than those who were not vaccinated [22]. Among the same cohort of students a follow-up to month 24 visit was done to assess the effect of helminth and malaria exposure on the immune response. They found no reduction in HPV-16 or HPV-18 antibodies among girls who had evidence of helminth or malaria exposure (Nakalembe et al. [23]), which was similar to the Tanzanian studies. However, in the Ugandan study, malaria exposure was measured as opposed to active infection in the Tanzanian study [23].

Immunogenicity data based on 7 months geometric mean titers to Anti-HPV-16/18 was compared between studies that were purely in a LMIC (Africa [14] and India [19]) as well Europe [24] (Fig. 1). The results are comparable across the different populations in these studies (Fig. 1).

In conclusion, all together, the studies that have been conducted so far within diverse populations of the LMIC have demonstrated strong immunogenicity of the prophylactic HPV vaccines among girls, young and old women.

### Studies that assessed virological and disease end points

Three trials that assessed vaccine efficacy against virological and disease end point in addition to safety and immunogenicity were found where a LMIC had participated. The Philippines and Guetamala were the only LMICs that participated in these studies. Perez et al. [15] (Guetamala) in the trial described for immunogenicity above, carried out analyses of the quadrivalent vaccine efficacy in the per protocol efficacy population (PPE). This population included all subjects aged 16–24, received all three vaccine doses and were seronegative as well as PCR-negative for the relevant HPV type(s) at enrollment and PCR-negative at month 7 to the vaccine-related HPV types. They concluded that among vaccinated subjects in the per-protocol population from Latin America, the quadrivalent HPV vaccine was 92.8 and 100 % effective in preventing CIN and external genital lesions related to vaccine HPV types, respectively [15].

Muñoz et al. [25] conducted a multicentre, parallel, randomized, placebo-controlled, double-blind study among 24–45 years old women from seven countries including the Phillipines, the only LMIC in the group(*n* = 3819; 38 study centers in Colombia, France, Germany, Philippines, Spain, Thailand, and the USA). Women were randomized to receive placebo (*n* = 1911) or the quadrivalent HPV vaccine (*n* = 1908) in order to assess the safety, immunogenicity and efficacy of the quadrivalent HPV vaccine. HPV-6/11/16/18 related incidence of infection of at least 6 months’ duration and cervical and external genital disease (including cervical, vulvar, or vaginal intraepithelial neoplasia; adenocarcinoma in situ; cervical, vulvar, or vaginal cancer; and genital warts) were measured for efficacy. In the per-protocol population (no infection at baseline) efficacy against disease or infection related to HPV-6/11/16 and 18 was 90.5 % (95 % CI: 73.7–97.5) (4 of 1615 cases in the vaccine group *versus* 41/1607 in the placebo group) and 83 · 1 % (95 %CI: 50.6–95.8) (four of 1601 cases *versus* 23/1579 cases) against disease or infection related to HPV 16 and 18 alone. In the intention-to-treat population (infection present at baseline) efficacy against HPV-6/11/16/and 18 endpoint was 30 · 9 % (95 % CI: 11.1–46.5) (108/1886 cases *versus* 154/1883 cases) and against HPV-16/18 was 22 · 6 % (95 %CI 2 · 9−41 · 9) (90/1886 cases *versus* 115/1883 cases), since infection and disease were present at baseline [25]. In this study, Muñoz et al. found that the quadrivalent HPV vaccine was efficacious in women aged 24–45 years who were not infected with vaccine HPV types at enrolment.

Currently, there is an on-going phase 3, multinational, double-blind, and randomized controlled trial with the bivalent vaccine, involving 13 countries, with the Philippines as the only participating LMIC (Australia, Canada, Mexico, the Netherlands, Peru, Philippines, Portugal, Russia, Singapore, Thailand, the UK, and the USA) named Vaccine Immunogenicity and Efficacy study (VIVIANE) Skinner et al. [26]. The main objective of the VIVIANE trial is to assess immunogenicity, efficacy and safety of the HPV 16/18 AS04-adjuvanted vaccine among women 26 years or older [26]. Interim results at mean follow up of 40.3 months are similar to Muñoz et al. 2009 study and have shown that the HPV 16/18 vaccine is efficacious against infections and cervical abnormalities associated with the vaccine types, as well as infections with the non-vaccine HPV types 31 and 45 [26] among women ≥ 26 years.

Individual country analysis of efficacy end points in these studies was not done most probably due to inadequate sample size. Therefore, conclusions of efficacy end points from participating countries individually may not be drawn.

### Safety of the HPV vaccines

Safety of the HPV vaccines was also assessed in most of the studies described above (summarized Table 2). In most of these studies, assessments for safety were based on both solicited and unsolicited local symptoms like pain and swelling at injection site as well systemic symptoms like arthralgia, fatigue, fever, gastrointestinal symptoms, headache, myalgia, rash, or urticaria (Table 2).

In some of the studies, new onset of chronic diseases (NOCDs), medically significant conditions (MSCs), pregnancies and their outcomes were also assessed [14, 16, 17, 20]. In all these studies (Table 2), the HPV vaccines were found to be generally safe and well tolerated among the participants. Injection site pain was the most common complaint which appeared more frequently among the vaccine recipients in most of the studies while in others there was no apparent difference in the incidence of AEs between the vaccine and non vaccine groups (Table 2). In all the studies, there were no vaccine related serious adverse events (Table 2).

### Alternate dose schedules

Neuzil et al. [27] in Vietnam conducted an Open-label, cluster randomized, non-inferiority study assessing four schedules of the quadrivalent HPV vaccine delivered in 21 schools to 903 adolescent girls (aged 11–13 years at enrollment) to determine the immunogenicity of different dosing schedules of quadrivalent HPV vaccine. The schedules were the standard dosing schedule (at 0, 2, and 6 months) and three alternative dosing schedules (at 0, 3, and 9 months; at 0, 6, and 12 months; or at 0, 12, and 24 months. They found that among this population of adolescent girls in Vietnam, administration of the quadrivalent HPV vaccine on standard and alternative schedules was immunogenic and well tolerated. The use of two alternative dosing schedules (at 0, 3, and 9 months and at 0, 6, and 12 months) compared with a standard schedule (at 0, 2, and 6 months) did not result in inferior antibody concentrations [27]. Pre-specified non-inferiority was not met for the alternative dose schedule group receiving doses at 0, 12, and 24 months. In the same cohort of girls, LaMontagne et al. [28] conducted a follow up study and reported results at 29–32 months after the third dose to investigate whether the immune responses using three alternative dosing schedules (0, 3, 9 months; 0, 6, 12 months; or 0, 12, 24 months) were non-inferior to the standard schedule at >2 years after vaccination.

LaMontagne et al. found similar antibody concentrations at ≥29 months after three doses of HPV vaccine regardless of dose-timing, and that extended schedules did not produce inferior immune responses [28]. The findings also suggested that two doses of HPV vaccine delivered at 0 and 12 months might afford similar protection.

In Uganda, LaMontagne et al. [29] investigated the immunogenicity of bivalent HPV vaccines among adolescent girls at mean age of 10 years who received one, two, or three vaccine doses in a demonstration project. They measured HPV16- and HPV18-specific antibodies at ≥24 months after the last vaccine dose using an enzyme linked immunoassay in girls who received one (*n* = 36), two (*n* = 145), or three (*n* = 195) doses. They found that even though immunogenicity with less than three doses did not meet a priori non-inferiority thresholds, antibody levels measured at ≥24 months after last dose were similar to those of adult women who had been followed for more than eight years for efficacy [29].

In conclusion, alternative dose schedules have shown non inferior immunogenicity among the 10–13 year old age group when compared to standard dosing schedules in populations where they have been evaluated.

### Unpublished, ongoing and stopped HPV vaccine studies in LMICs

In addition to the published clinical trials from LMICs in this review, a total of ten unpublished ongoing, suspended, withdrawn or completed clinical trials were found (clinicalTials.gov; accessed 4th May 2015) within LMIC. India had the largest number (8/10) with six of them using qHPV while two were using bHPV. The qHPV Indian trials are mainly safety and immunogenicity studies among HIV populations (two of them), healthy populations (two of them; one suspended) while another was a vaccine delivery strategy study which was withdrawn. Likewise, the two bHPV vaccine studies are assessing safety and Immunogenicity among HIV positive and negative populations (clinicalTrials.gov accessed 4th May 2015).

However, in India, there were media allegations that HPV vaccines caused death of four girls in Northern India. Acting on this information, the government of India suspended two HPV vaccine studies and instituted an inquiry into the safety of both vaccines [30]. One study was a clinical trial to investigate the immunogenic efficacy of two doses (6 months apart) compared with the three doses (0, 2, 6 months) of Gardasil while the second one was a feasibility study to assess a school-based and community-based vaccination program [30]. The inquiry concluded that no deaths were related to the vaccine [31].

In addition to the Indian studies, two safety and immunogenicity studies were found among pre adolescents in Kenya; HIV positive and negative populations (clinicalTrials.gov accessed 04th May 2015). Results from these trails are yet to be published.

## Discussion

We have reviewed results of safety, immunogenicity, efficacy (virological and disease end points) for both the bivalent and quadrivalent HPV vaccine in LMIC. This review identified ten published clinical trials that involved a LMIC. According to the clinicalTrials.gov (accessed 23rd March 2015), a total of 159 cervarix or gardasil clinical trials have so far been registered, ongoing or completed. Both the published and unpublished clinical trials within the LMICs trials are still few when compared to the HICs. The discrepancy may in part be due to ethical, scientific and logistical challenges that face the conduct of clinical research in LMIC [32].

In this review, we found that only five HPV vaccine studies (one clinical trial; two longitudinal observational studies nested within the clinical trial; two cross sectional studies) have been conducted within the LMICs of Sub-Saharan Africa (SSA), yet, SSA has a high burden of cervical cancer. Additionally, SSA is one of the countries with a high incidence of penile cancers, though overall, its global incidence is low [4]. SSA has prevalent potentially immune modulating situations (Malaria, Helminths, HIV and malnutrition) that may affect the immune response and finally efficacy of the HPV vaccines [6]. However, the few studies so far have demonstrate that the HPV vaccines are highly immunogenic and safe, though there is need for population based studies designed to address long term effectiveness of the HPV vaccines based on the virological and disease end points.

All the studies in this review that investigated immediate vaccine safety did not record a serious adverse event related to the vaccine. Serious adverse events that have been reported as potential signals have not been confirmed after detailed investigations [33]. The safety results from these studies are similar to what has been concluded from reviews of long term safety that included studies from HICs [34]. However, much as the safety reports are so far reassuring, as with any new vaccine, post marketing surveillance mechanisms should be in place to monitor long-term safety [35].

Published vaccine efficacy studies that were found in this review where a LMIC participated were multi center studies. Currently, we identified only six clinical trials with cervarix or gardasil that are ongoing or completed within a LMIC (ClinicalTrials.gov accessed 23rd March 2015). Notably in Sub-Saharan Africa, two Phase three randomized clinical trials that were found (clinical Trials.gov accessed 23rd March 2014) are all from Kenya. The trials are assessing immunogenicity to the qHPV vaccine among HIV-infected adolescents (9–14) while one of them is assessing safety, tolerability and immunogenicity of the qHPV vaccine among healthy women 9–26 years. The other four ongoing or completed trials with cervarix or gardasil are also assessing safety and immunogenicity among healthy and HIV infected Indian populations as well as alternative dose regimens. Results from these trials which are yet to be posted will not feature vaccine efficacy as this was not part of the objectives. Therefore, HPV vaccines efficacy studies within the LMICs should be considered.

The few clinical trials that were conducted within the LMIC demonstrated that the 9–15 year old age group mounted a higher immune response when compared to the older age groups (above 15 years). This finding was consistent with what was observed in other clinical trials that were conducted elsewhere [36]. Further, efficacy among LMIC adult women (>25 years) populations was demonstrated as well as cross protective potential as has been observed in other studies [7].

After evidence of non-inferiority of two-dose schedule when compared to the three-dose schedule from clinical trials, WHO currently recommends a two dose schedule at 6-months interval of the bivalent or quadrivalent vaccine for girls vaccinated before age 15 years and a three dose schedule for those aged ≥15 years or those with immune compromising conditions [33]. On alternative dose regimens, the only published clinical trial in this review assessed different schedules of the three doses instead of two doses [27]. For LMIC, it is more important to have studies that assess less dose regimens instead of alternative three dose schedules as it is of critical importance to save resources on any extra dose administered. The only published study that investigated less than three doses of the bivalent HPV-16/18 vaccine was not a randomized controlled trial of 2 *versus* three dose regimen and had a small sample size and therefore was not powered to test the primary hypothesis of non-inferiority. Nevertheless, this study still suggests that even with less than three doses, antibody levels were still comparable with older women were efficacy has been proven [29]. However, it remains to be known for how long the level of response from the two doses would last.

Second generation vaccines are being considered to address some of the limitations faced by LMIC with the current prophylactic vaccines, which include limited valence and dependency on the cold chain among others [7]. So far, Merck Inc. has developed a nine valent HPV vaccine, which target five additional oncogenic HPV types 31, 33, 45, 52 and 58 to 6, 11, 16 and 18. This vaccine has been approved and registered by the FDA for use in girls and young women 9 to 26 years of age for the prevention HPV 16, 18, 31, 33, 45, 52 and 58 related cervical, vulvar, vaginal, and anal cancers as well as genital warts caused by HPV types 6 and 11 [37]. Worldwide, HPV-16/18 contribute to about 70 % of cervical cancer cases while HPV-31/33/45/52/58 a further 20 % [38]. Therefore, this vaccine has the potential to prevent 90 % of cervical cancer disease worldwide since the estimates of the 9-HPV types in cervical cancer do not significantly differ across the world [39]. However, a review on Human papillomavirus distribution in invasive cervical carcinoma in sub-Saharan Africa found that the relative contribution of HPV-45 to ICC was significantly higher in SSA when compared to the rest of the world [40]. In this study, it was also found that HPV18 was higher and HPV45 lower in HIV endemic areas. However, the significance of this finding remains to be understood in light of emerging evidence that HPV vaccines are immunogenic even in HIV infected individuals despite need for more long term data [41]. Further, Ndiaye et al. highlighted the importance of HPV-45 as an important candidate for future vaccines, despite the fact that varying degrees of cross protection against HPV45 have been described for the current bivalent and quadrivalent vaccines [38]. Since the level of potential benefit of the nine-valent vaccine may be attenuated by the cross protection properties of the previous vaccines, the benefits must be weighed against the cost of the vaccines [42] especially in LMIC. However with no ongoing studies to assess the current vaccines impact over time, it will be difficult to analyse the cost effectiveness of the second generation vaccines in the LMIC. In addition, decisions to revaccinate with the new HPV vaccines those who are already vaccinated with the older vaccines will be difficult without this evidence.

## Conclusion

The few studies in this review that assessed safety, Immunogenicity and efficacy of the HPV vaccines in the LMIC had a similar safety and Immunogenicity profile like in high income countries. Compared to high income countries, there are much fewer studies on safety and immunogenicity conducted in LMIC and the number of study participants is also lower, so data from LMIC is still limited. Notably, HPV vaccine efficacy data is largely lacking with no efficacy study specifically identified for a LMIC though the available studies have provided evidence of efficacy. Specifically, in Sub-Saharan Africa where immune compromising/modulating situations are prevalent there is need for more robust better designed long term immunogenicity and effectiveness studies [6, 43].

Overall, there is need for evidence of the long term clinical effectiveness and duration of protection after two dose regimens as is elsewhere [35]. This evidence will also help to guide decisions on the second generation vaccines.

The review highlighted the HPV vaccine published trials within the LMIC. The identified research needs are in line with WHO recommendations for future research needs.

## Abbreviations

HPV: Human papilloma virus
ELISA: Enzyme-linked immunosorbent assay
VLP: Virus like particles
GMTs: Geometric mean titers
cLIA: Competitive Luminex immunoassay
LMICs: Low middle income countries
HICs: High income countries
WHO: World Health Organisation
GAVI: Global alliance for vaccine initiative
SSA: Sub-Saharan Africa
ICC: Invasive cervical cancer
HIV: Human immunodeficiency syndrome
